# Edible Bird’s Nest Protects Against Hyperglycemia-Induced Oxidative Stress and Endothelial Dysfunction

**DOI:** 10.3389/fphar.2019.01624

**Published:** 2020-02-04

**Authors:** Dharmani Devi Murugan, Zuhaida Md Zain, Ker Woon Choy, Nor Hisam Zamakshshari, Mel June Choong, Yang Mooi Lim, Mohd Rais Mustafa

**Affiliations:** ^1^ Department of Pharmacology, Faculty of Medicine, University of Malaya, Kuala Lumpur, Malaysia; ^2^ Department of Anatomy, Faculty of Medicine, Universiti Teknologi MARA, Sungai Buloh, Malaysia; ^3^ Centre for Natural Product Research and Drug Discovery (CENAR), Wellness Research Cluster, University of Malaya, Kuala Lumpur, Malaysia; ^4^ Centre for Cancer Research, Faculty of Medicine and Health Sciences, University Tunku Abdul Rahman, Selangor, Malaysia; ^5^ Department of Pre-clinical Sciences, Centre for Cancer Research, Faculty of Medicine and Health Sciences, University Tunku Abdul Rahman, Selangor, Malaysia

**Keywords:** hydrolyzed bird’s nest, hyperglycemia, oxidative stress, endothelial dysfunction, reactive oxygen species, nitric oxide

## Abstract

Increased oxidative stress by hyperglycemia is a major cause of vascular complications in diabetes. Bird’s nest, which is made from the saliva of swiftlets has both medicinal and nutritional values dated back to ancient China. However, its role in improving endothelial dysfunction due to diabetes is yet to be elucidated. The present study examined the protective effect and mechanism of action of the aqueous extract of hydrolyzed edible bird nest (HBN) on endothelium in models of diabetes, *in vitro* and *in vivo*. Male *db/m+* and *db/db* mice were orally administered with or without HBN and glibenclamide for 28 days, followed by vascular reactivity studies in mouse aortas. Human umbilical vein endothelial cells (HUVECs) and isolated mouse aorta from C57BL/6J were treated with high glucose (HG), HBN, sialic acid (SA), glibenclamide, and apocynin, respectively. The effects of HBN on reactive oxygen species (ROS) production and nitric oxide (NO) bioavailability were assessed by Western blot, 2′,7′-dichlorofluorescin-diacetate (DCF-DA), and 4-amino-5-methylamino-2′,7′ difluorofluorescein (DAF-FM DA) in HUVECs, isolated mouse aorta, and *db/db* diabetic mice. HBN significantly reversed the endothelial dysfunction in diabetic mice and isolated mouse aorta. HBN normalized ROS over-production of NOX2 and nitrotyrosine, reversed the reduction of anti-oxidant marker, SOD-1 as well as restored NO bioavailability in both HUVECs challenged with HG and in *db/db* diabetic mice. Similarly, HG-induced elevation of oxidative stress in HUVECs were reversed by SA, glibenclamide, and apocynin. This attests that HBN restores endothelial function and protects endothelial cells against oxidative damage induced by HG in HUVECs, isolated mouse aorta, and *db/db* diabetic mice *via* modulating ROS mechanism, which subsequently increases NO bioavailability. This result demonstrates the potential role of HBN in preserving endothelial function and management of micro- or macrovascular complications in diabetes.

**Graphical Abstract:**
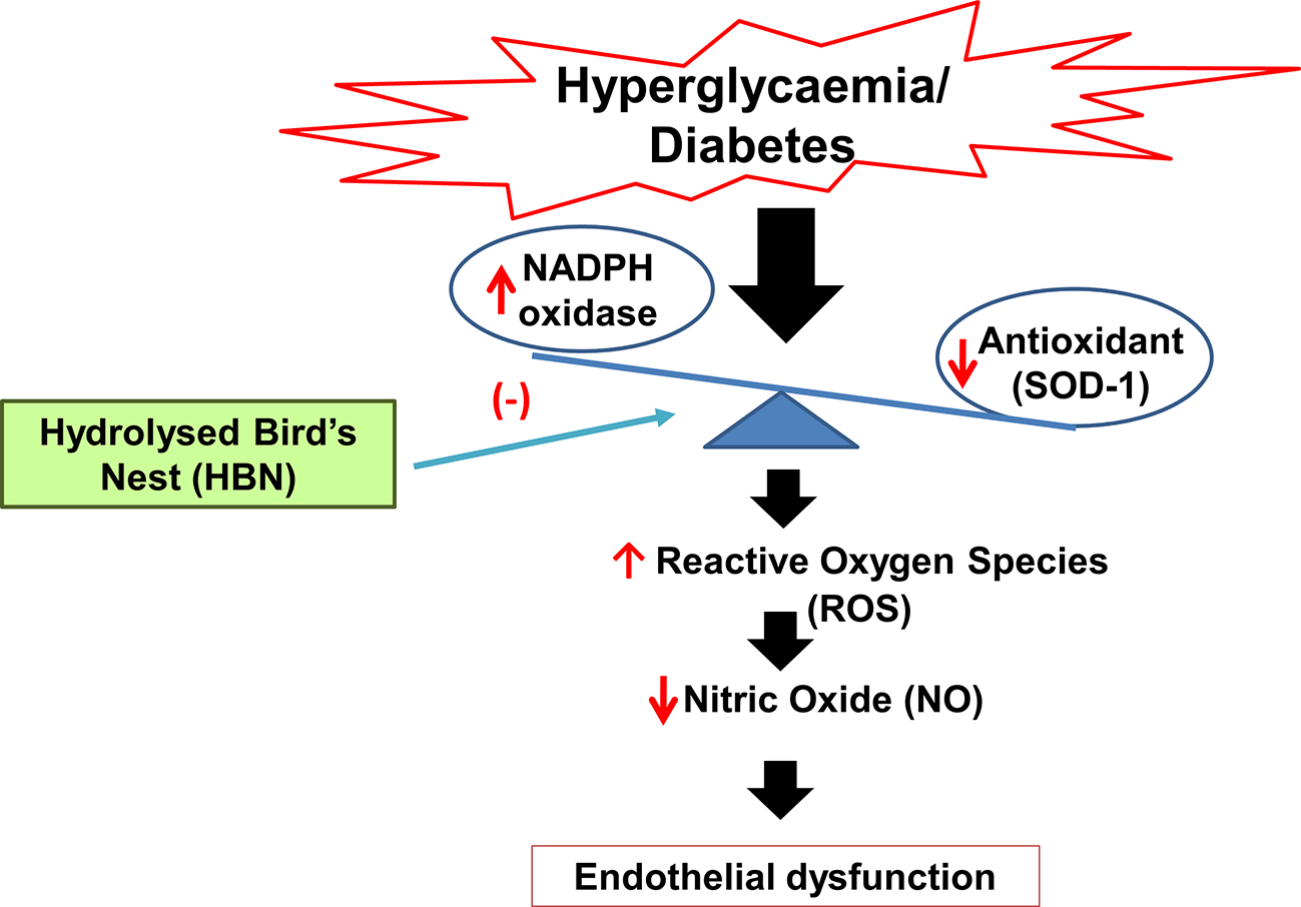
Mechanism of action of hydrolyzed bird’s nest on hyperglycemia-induced endothelial dysfunction.

## Introduction

The endothelium plays a pivotal role in physiology and pathophysiology of the vasculature, including modulating the vascular tone, cellular adhesion, smooth muscle, cell proliferation, thromboresistance, and inflammation of vessel wall (Sena et al., 2013). Under normal physiological condition, the healthy endothelium maintains a fine balance between vasoconstriction and vasodilatation factors (Sena et al., 2013). Nitric oxide (NO) is among the endothelium-derived factor which controls the vasodilatory effect whereas factors such as reactive oxygen species (ROS), thromboxane, and endothelin 1 exerts vasoconstrictor effects (Just et al., 2008; Loscalzo, 2013). Endothelial dysfunction is commonly associated with increased cellular oxidative stress and with decreased nitric oxide (NO) bioavailability, resulting in dysregulation of vascular tone and ultimately compromised cardiovascular function (Pitocco et al., 2010). Endothelial dysfunction is correlated with various cardiovascular and metabolic diseases such as hypertension, atherosclerosis, and diabetes mellitus (Sena et al., 2013).

Over the years, hyperglycemia-induced oxidative stress has been known as a key process in the onset of diabetic complications (Matough et al., 2012). Hyperglycemia causes excessive production of ROS, especially superoxide anions (O_2-_) which are generated through partial reduction of molecular oxygen to O_2-_ by NADPH oxidase, uncoupled endothelial nitric oxide synthase (eNOS), mitochondrial electron transport chain as well as xanthine oxidase (Jay et al., 2006). O_2−_ will react with NO to form the toxic peroxynitrite (ONOO−), which uncouples eNOS to produce more superoxide anions. This vicious continuous cascade of events reduces NO bioavailability and leads to endothelial dysfunction (Schulz et al., 2008).

The edible bird’s nest is made from the saliva of swiftlets inhabiting the limestone caves. Edible bird’s nest has been used in Chinese cuisine mainly in the form of bird’s nest soup since 1,200 years ago as it is believed to enhance energy levels, prevent aging, and improve overall well-being (Ma and Liu, 2012). Edible bird’s nest has lethal dose (LD_50_) cut off more than 5,000 mg/kg and is classified as category 5 or unclassified category of globally harmonized classification system (GHS), therefore it is safe to be taken by to humans (Haghani et al., 2016). Furthermore, there are scientific reports of its anti-oxidative, anti-inflammatory, influenza virus inhibitory effect, hemagglutination-inhibitory activities, and bone-strengthening eﬀects (Kong et al., 1987; Guo et al., 2006; Matsukawa et al., 2011; Ma and Liu, 2012; Vimala et al., 2012; Yida et al., 2014). In 2015, Yida et al. showed that edible bird’s nest prevents high-fat diet- (HFD) induced insulin resistance in rats. However, thus far, the protective effect of edible bird’s nest in glucotoxicity condition has not been studied. Therefore, the present study investigated the effect of hydrolyzed bird’s nest (HBN) in abating oxidative stress and improving endothelial dysfunction in the hyperglycemia-induced oxidative stress in models of diabetes, *in vitro*, *ex vivo*, and *in vivo*.

## Materials and Methods

### Quantification of Sialic Acid in Hydrolyzed Bird’s Nest

The HBN was prepared and kindly provided by Professor YL from University Tunku Abdul Rahman, Malaysia. A voucher specimen was deposited in the Nature Inspired UM Natural Products Library, University Malaya (voucher number UMCNA1801). Briefly, the raw edible birds nest sample eluted in distilled water was boiled at 80°C for an hour. The extracts were filtered with membrane filter paper and the resulting filtrates were lyophilized and stored at −80°C for further use. Liquid chromatography-mass spectrometry quadrupole–time of flight (LCMS Q-TOF) was used to determine the amount of sialic acid, one of the active compounds in HBN. The LCMS Q-TOF protocol was adapted from Marni et al., 2014 with slight modification (Marni et al., 2014). Briefly, a series of a standard solution of sialic acid with different concentration (50, 25, 12.5, 6.3, 3.1, and 1.5 µg/mL) and 10 mg/mL of HBN were prepared in distilled water. The samples were sonicated for 10 min and were subjected for LCMS Q-TOF analysis. The C-18 reversed phased column with diameter 4.6 × 250 mm was used with a mobile phase of water: methanol (1:1), the flow rate of 2 mL.min-1, sample quantity of 10µL, and column temperature 25° C. HBN and standard sialic acid were run in triplicates. A linear graph was plotted using standard sialic acid and the sialic acid content in HBN was determined from the standard curve. A standard solution graph of concentration versus area was plotted. Finally, the sialic acid concentration of each extract was determined based on the graph.

### Animals

Male *db/m+* and *db/db* mice (10 weeks old) were obtained from The Jackson Laboratories (Bar Harbor, ME, USA) for the *in vivo* experiment while male C57BL/6J (12 weeks old) mice were obtained from the Monash University (Sunway Campus, Malaysia) for the *ex vivo* experiments. All the experimental procedures were approved by the University of Malaya Animal Care and Ethics Committee (Ethics Reference No: 2015-180709) and accredited by Association for Assessment and Accreditation of Laboratory Animal Care International (AAALAC). Animal study was carried out in strict accordance with the established institutional guidelines and the NIH guidelines on the use of experimental animals. The animals were housed in a well-ventilated room maintained at a temperature of 23°C with 12 h light/dark cycles, 30–40% humidity and had free access to standard rat chow (Specialty Feeds Pty Ltd., Glen Forrest, Australia) and filtered tap water.

### *Ex Vivo* Culture of Mouse Aortic Rings

The male C57BL/6J mice were euthanized using carbon dioxide (CO_2_), and the aorta was carefully isolated and immersed in sterile phosphate buffer saline (PBS). The aortas were cleaned from fat and connective tissues under the microscope and cut into several segments of approximately 2 mm in length. The aortic rings were incubated in normal glucose (NG, 2.5 mM) or high glucose (HG, 30 mM) with or without co-incubation of HBN (15 and 30 μg/mL), sialic acid (20 μg/mL), glibenclamide (10 μM), apocynin (20 μM), and compound C for 48 h at 37°C in Dulbecco’s Modified Eagle’s Media (DMEM; Gibco, Gaithersburg, MD, USA) with 10% fetal bovine serum (FBS; Gibco), 100 µg/mL streptomycin, and 100 U/mL penicillin (Lau et al., 2013). The concentrations of HBN used in this study were determined using MTT assay (data not shown).

### HBN Treatment in *db/db* Mice

The male *db/db* mice male randomly assigned into four groups (n = 6 per group) of mice receiving: (a) vehicle (distilled water); (b) hydrolyzed bird’s nest (75 mg/kg); (c) hydrolyzed bird’s nest (150 mg/kg); (d) glibenclamide (1mg/kg) by oral gavage for four weeks. The *db/m+* mice (n = 6) was used as the non-diabetic control. The animals were humanely sacrificed by CO_2_ inhalation at the end of treatment. Blood samples were collected from inferior vena cava after an overnight fast, and the serum were stored at −80°C for total nitrate/nitrite assay. The aorta was excised and cleaned of adjacent connective tissues and fat and cut into rings for functional studies and some arteries were snap-frozen in liquid nitrogen and stored at −80°C for further experiments.

### Functional Study

The aortic rings from the treated groups and organ-cultured rings were mounted on myograph chamber containing 5 mL of oxygenated Krebs solution which consists mM of NaCl 119, NaHCO_3_ 25, KCl 4.7, KH_2_PO_4_ 1.2, MgSO_4_·7H_2_O 1.2, glucose 11.7, and CaCl_2_.2H_2_O 2.5. The aortic rings were maintained at 37°C and stretched to optimal baseline tension of 3 millinewtons (mN) in a Multi-Wire Myograph System (Danish Myo Technology, Aarhus, Denmark) and continuously oxygenated with 95% O_2_ and 5% CO_2_. The changes of isometric tension of aortic rings in response to different drugs were recorded using the PowerLab LabChart 6.0 recording system (AD Instruments, Australia). The rings were equilibrated for approximately 45 min and pre-contracted with high 60 mM KCl solution followed by three times of washing with Krebs solution. Once the tension stabilized and returns to baseline, phenylephrine (3 µM) was added to induce contraction followed by generation of endothelium-dependent relaxation (EDR) by cumulative addition of acetylcholine (ACh) from 3 to 10 nM. The endothelium-independent relaxation (EIR) was generated by the addition of sodium nitroprusside (SNP) from 1 nM to 10 µM. Each experiment was conducted on separate rings from six mice. Concentration-response curves for relaxations were conveyed as the percentage of reduction in contraction induced by phenylephrine before the application of ACh or SNP. The maximum effect (*R*_max_) and concentration inducing 50% of *R*_max_ (pEC_50_) were determined from the cumulative concentration-response curves.

### Detection of Vascular Superoxide Formation

Lucigenin-enhanced chemiluminescence assay was used in this study to quantify the production of vascular superoxide anion. The organ-cultured rings from each group were incubated for 45 min at 37°C in Krebs-HEPES buffer (in mM: NaCl 99.0, NaHCO3 25, KCl 4.7, KH2PO4 1.0, MgSO4 1.2, glucose 11.0, CaCl 22.5, and Na-HEPES 20.0) in the presence of diethylthiocarbamic acid (DETCA, 1 mM) and β-nicotinamide adenine dinucleotide phosphate (β-NADPH, 0.1 mM). DECTA acts as an inactivator for superoxide dismutase (SOD) while β-NADPH acts as a substrate for NADPH oxidase. The NADPH oxidase inhibitor, diphenylene iodonium (DPI; 5 mM) was then added as a positive control. Before measurement, a solution containing lucigenin (5 mM) and β-NADPH (0.1 mM) in Krebs-HEPES buffer was added into each well of 96-well Optiplate. Background photo emissions were measured every 30 s for 20 min using Hidex plate CHAMELEONTM V (Finland). After addition of the rings into the wells, the measurement was taken again. The rings were then dried for 48 h at 65°C and weighed. The data were expressed as average counts per weight of dried vessel (mg) and was compared over the normal glucose (Choy et al., 2017).

### Cell Culture

Human umbilical vein endothelial cells (HUVECs, Lonza, Basel, Switzerland, No. CC-2517) were cultured in normal glucose endothelial cell medium (ECM) supplemented with 10% fetal bovine serum (FBS), 100 U/mL penicillin, 100 µg/mL streptomycin, and 50 µg/L endothelial cell growth supplement (all Sciencell, Carlsbad, CA). The cells were incubated in a humidified atmosphere containing 5% CO_2_ at 37°C. Cells from passages 5 to 9 were used for the current study. Experiments were performed once the cells reached 80% confluency. The cells were then starved for 4 h in FBS-free ECM before treatment with either normal glucose (5.5 mM, NG) and high glucose (HG, 30mM) co-treated with hydrolyzed bird’s nest HBN (30 μg/mL), sialic acid (20 μg/mL), glibenclamide (10 μM), and apocynin (20 μM) for 48 h. Another set was incubated with osmotic control, mannitol (25 mM) (Lau et al., 2013).

### Measurement of Intracellular ROS Generation

The amount of intracellular ROS generation in HUVECs was measured using DCF-DA fluorescein (Invitrogen, CA, USA) dye. ROS was detected after formation of fluorescent DCF product inside the cell due to oxidation of DCF-DA. In brief, 1 × 10^4^ HUVECs were seeded into 96 well plates. After overnight incubation in 5% CO_2_ at 37°C, the cells were starved and treated as described. Another set of cells were treated with H_2_O_2_ (200 µM), the ROS inducer 4 h before the end of 48 h incubation as a positive control. The treated cells were incubated for 48 h and the media was then removed. The wells were rinsed with phosphate buffered saline PBS followed by addition of 10 µM of DCF-DA into each well. The absorbance was then measured kinetically for 1 h using a fluorescent multimode reader (Infinite M1000 Pro; Tecan US, Morrisville, NC) at fluorescence excitation and emission of 492/517 nm. The data was presented as the fluorescent intensity (a.u.) at 50^th^ minute after addition of DCF-DA dye.

### Measurement of NO Production in HUVECs

The amount NO production in HUVECs was measured using DAF-FM diacetate (Invitrogen, CA, USA) dye (Namin et al., 2013). Basically, NO within the cells will react with DAF-FM diacetate to form fluorescent benzotriazole. The confluent HUVECs were seeded into 96 well plates and treated accordingly as described in the previous section. HUVECs treated with calcium ionophore (A23187, 5 µM) were used as positive control. After 48 h incubation, the media was removed and rinsed with PBS three times. Five micrograms of DAF dye was added into each well, and the absorbance was measured kinetically for 1 h using a fluorescent multimode reader (Infinite M1000 Pro; Tecan US, Morrisville, NC) at fluorescence excitation and emission of 495/515nm. The results were presented as a value of fluorescent intensity (a.u) at 50^th^ minute after addition of the dye.

### Measurement of Total Nitrite/Nitrate Levels

Total nitrite and nitrate level from the mice serum was detected using Nitrate/Nitrite Colorimetric Assay Kit (Cayman Chemical Company, Ann Arbor, MI, USA) according to the manufacturer’s protocol. Absorbance was measured using a plate reader (Tecan, Mannedorf, Switzerland) with an absorbance of 540nm. The results are expressed in µM.

### Western Blot

Protein samples from the treated mouse aorta and HUVECs were lysed in ice-cold 1X RIPA buffer consists of EGTA 1 mM, EDTA 1 mM, NaF 1 mM, leupeptin 1 µg/mL, aprotinin 5 µg/mL, PMSF 100 µg/mL, sodium orthovanadate 1 mM, and β-glycerolphosphate 2 mg/mL. The lysates were collected and centrifuged at 20,000 g for 20 min. Protein concentrations were determined using standard Lowry assay protocol by (Bio-Rad Laboratories, Hercules, CA, USA). Fifteen micrograms of protein samples were electrophoresed at 100 V through 7.5% or 10% SDS-polyacrylamide gels based on the size of target proteins and transferred to an Immobilon-P polyvinylidene difluoride membrane (Millipore, Billerica, MA, USA). The membranes were blocked from any non-specific binding by 3% bovine serum albumin (BSA) in 0.05% Tween 20 PBS with gentle shaking. The membranes were then incubated with primary antibodies against NADPH oxidase 2 (NOX-2; 1:1,000, Abcam), nitrotyrosine (1:1,000, Abcam) superoxide dismutase-1 (SOD-1; 1:1,000, Santa Cruz), phosphorylated endothelial nitric oxide synthase (p-eNOS) at Ser1177 (1:1,000, Abcam), endothelial nitric oxide synthase (eNOS) (1;1,000, BD Transduction laboratory, San Diego, CA, USA), and β-actin (1:10,000, Abcam) at 4°C overnight. Following incubation, the membranes were washed three times with TBS-T and incubated with horseradish peroxidase-conjugated secondary antibodies (DakoCytomation, Carpinteria, CA, USA) for 2 h at room temperature. Finally, the enhanced chemiluminescence detection system (ECL reagents, Millipore Corporation, Billerica, MA) was added onto the membrane and exposed on X-ray films. The films were then automatically processed and developed by SRX-101 (Konica, Wayne, NJ). The densitometry analysis was performed using Quantity One software (Bio-Rad). The respective protein expression levels for nitrotyrosine, NOX-2, and SOD-1 were normalized to β-actin, p-eNOS to eNOS, and then compared with control.

### Data Analysis

Results are presented as mean ± SEM from n experiments. Concentration-response curves were fixed to a sigmoidal curve using non-linear regression using the statistical software GraphPad Prism version 4 (GraphPad Software Inc., San Diego, CA, USA). Statistical significance was determined using two-tailed Student’s t-test for comparison of two groups and a one-way ANOVA followed by Bonferroni multiple comparison tests for comparisons of more than two groups. *P* < 0.05 was considered statistically significant.

## Results

### Sialic Acid Content in Hydrolyzed Bird’s Nest

**Figure 1:**
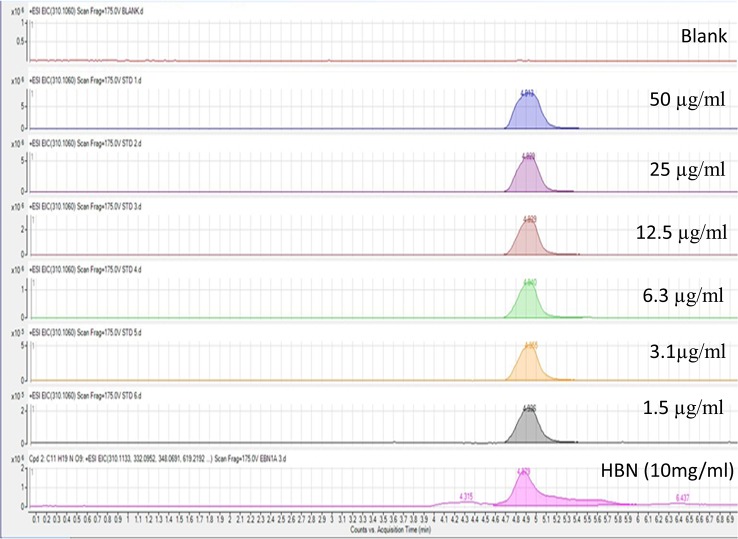
Liquid chromatography-mass spectrometry spectrum of standard sialic acid at different concentrations and 10 mg/mL hydrolyzed bird nest (HBN). HBN contained 1.26 µg sialic acid/mg HBN based on their molecular weight and retention time.

Six difference standards of sialic acid (N-acetylneuraminic acid) were run on the LCMS Q-TOF and result was plotted. The standard graph for sialic acid was obtained from the calibration equation *y* = (3 × 10^6^) *x* + 532,337 (*R* ^2^ = 0.9931) where y is the peak area and *x* is the weight of sialic acid content in the extract. The LCMS spectrum for standard together with HBN is shown in **Figure 1**. Sialic acid appeared at the retention time of 4.9 min of the LCMS spectrum. The amount of sialic acid in the sample was determined based on the molecular weight of 309.107 g/mol and their retention time. From this analysis, it shows that HBN contained 1.26 µg sialic acid/mg.

### HBN Ameliorated Endothelial Dysfunction in Mouse Aorta

**Figure 2:**
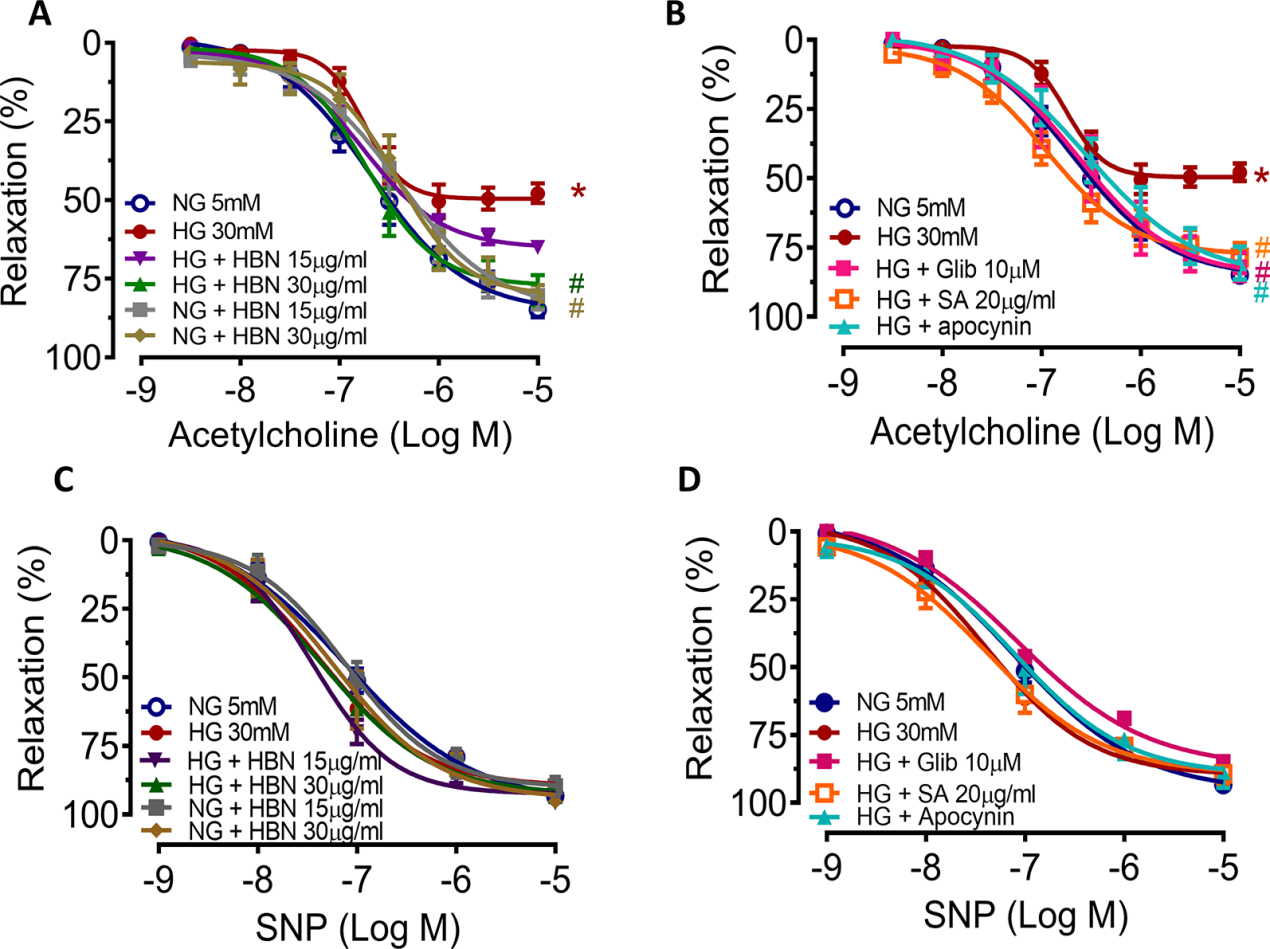
The effect of isolated aorta from C57BL/6J treated with normal glucose (NG, 5 mM), high glucose (HG, 30 mM), hydrolyzed bird nest (HBN, 15 and 30 μg/mL), sialic acid (SA, 20 μg/mL), glibenclamide (Glib, 10 μM), and apocynin (20 μM) for 48 h in acetylcholine-induced endothelium-dependent relaxation (EDR) **(A, B)** and sodium nitroprusside-induced endothelium-independent relaxation (EIR) **(C, D)**. Results are means ± SEM of six experiments. *P < 0.05 compared with NG, ^#^P < 0.05 when compared with HG.

To determine the role of HBN treatment in high glucose-induced endothelial dysfunction in mice, we examined EDR and EIR in response to ACh and SNP respectively in a concentration-dependent manner. Aorta from mice treated with HG for 48 h displayed 48% relaxation to ACh-induced relaxation compared to control group which showed 85% relaxation. Co-treatment with HBN (15 and 30 μg/mL) restored the impaired relaxation to ACh in a concentration-dependent manner, with HBN at 30 μg/mL being an effective concentration in the HG-treated aorta. HBN alone did not affect the ACh relaxation in the NG-treated aorta (**Figure 2A** and **Table 1**). Additionally, co-incubation with sialic acid (20 μg/mL), glibenclamide (10 μM), and apocynin (20 μM) reversed the HG-induced impairment of relaxation to ACh (**Figure 2B** and **Table 1**). SNP-induced relaxations were similar in all groups, reflecting the lacked changes in the sensitivity of vascular smooth muscle to NO (**Figures 2C–D** and **Table 1**).

**Table 1:** Agonist sensitivity (pEC_50_) and % maximum relaxation (*R*_max_) of acetylcholine (ACh)-induced endothelium-dependent relaxation and sodium nitroprusside (SNP)-induced EIR in isolated aorta from C57BL/6J mice treated with normal glucose (NG, 5 mM), high glucose (HG, 30 mM), hydrolyzed bird nest (HBN, 15 and 30 μg/mL), sialic acid (SA, 20 μg/mL), glibenclamide (Glib, 10 μM), and apocynin (20 μM) for 48 h.

Groups	ACh		SNP	
	pEC_50_ (log M)	*R*_max_ (%)	pEC_50_ (log M)	*R*_max_ (%)
NG	−6.69 ± 0.10	84.88 ± 2.48	−7.11 ± 0.12	93.30 ± 1.52
NG + HBN (15µg/mL)	−6.40 ± 0.19	80.26 ± 4.58	−7.12 ± 0.14	89.86 ± 3.46
NG + HBN (30µg/mL)	−6.42 ± 0.11	79.73 ± 2.64	−7.23 ± 0.18	95.24 ± 2.55
HG	−6.75 ± 0.07	47.92 ± 3.12^*^	−7.39 ± 0.13	91.53 ± 2.07
HG + HBN (15µg/mL)	−6.76 ± 0.06	64.97 ± 1.70	−7.44 ± 0.09	95.57 ± 1.25
HG + HBN (30µg/mL)	−6.73 ± 0.09	79.10 ± 5.14^#^	−7.35 ± 0.17	93.38 ± 2.34
HG + Glib	−6.63 ± 0.18	82.13 ± 5.03^#^	−7.08 ± 0.21	84.87 ± 1.70
HG + SA	−6.95 ± 0.14	78.67 ± 5.18^#^	−7.38 ± 0.20	89.33 ± 3.89
HG + Apocynin	−6.54 ± 0.17	80.68 ± 5.93^#^	−7.09 ± 0.23	88.77 ± 5.85

Results are means ± SEM (n = 6). *P < 0.05 compared with NG, ^#^P < 0.05 when compared with HG.

**Figure 3:**
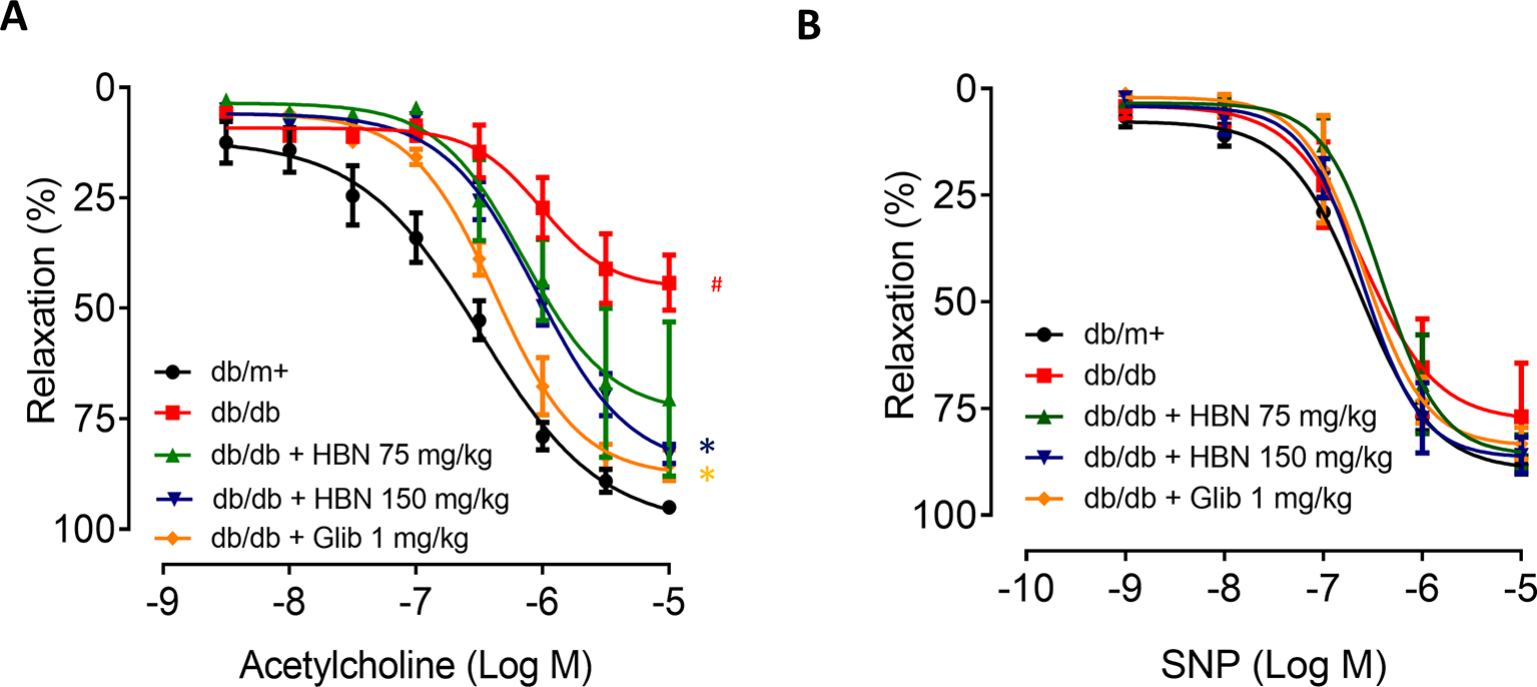
The effect of four week’s treatment of hydrolyzed bird nest (HBN, 75 and 150 mg/kg) and glibenclamide (1 mg/kg) on **(A)** acetylcholine-induced endothelium-dependent relaxation (EDR) and **(B)** sodium nitroprusside-induced endothelium-independent relaxation (EIR) in db/db mice. Results are means ± SEM of six experiments. # P < 0.05 compared with db/m+, *P < 0.05 when compared with db/db.

The maximal relaxation and sensitivity to ACh in the aorta from *db/db* mice treated with vehicle was significantly lesser compared to the non-diabetic group (*R*_max_: 57.04 ± 3.29% vs. 95.03 ± 1.31%, respectively). Four-weeks treatment with HBN (150 mg/kg) and glibenclamide (1 mg/kg) reversed the impaired ACh- induced relaxation in *db/db* aorta (**Figure 3A** and **Table 2**) while no significant changes was observed in SNP-induced relaxation (**Figure 3B** and **Table 2**).

**Table 2:** Agonist sensitivity (pEC50) and % maximum relaxation (*R*_max_) of acetylcholine-induced endothelium-dependent relaxation and sodium nitroprusside (SNP)-induced EIR in isolated aorta from *db/db* mice treated with glibenclamide (1 mg/kg), HBN (75 mg/kg) and HBN (150 mg/kg).

Groups	Ach		SNP	
	pEC_50_ (log M)	*R*_max_ (%)	pEC_50_ (log M)	*R*_max_ (%)
*db/m*+	−6.49 ± 0.11	95.03 ± 1.31	−6.62 ± 0.08	88.27 ± 2.12
*db/db*	−5.93 ± 0.15 ###	57.04 ± 3.29	−6.59 ± 0.28	76.91 ± 12.49
*db/db* + HBN 75mg/kg	−6.19 ± 0.22	70.56 ± 17.45	−6.41 ± 0.16	85.16 ± 3.92
*db/db* + HBN 150 mg/kg	−6.05 ± 0.07 ***	83.01 ± 2.17	−6.61 ± 0.13	85.98 ± 4.20
*db/db* + glibenclamide 1 mg/kg	−6.37 ± 0.06 ***	85.55 ± 3.48	−6.59 ± 0.17	83.21 ± 3.71

Results are means ± SEM (n = 6). ### *P* < 0.001 compared with *db/m+*, *** *P* < 0.001 when compared with *db/db*.

### HBN Reduced High Glucose-Induced Vascular Superoxide Production and Intercellular ROS Formation

**Figure 4:**
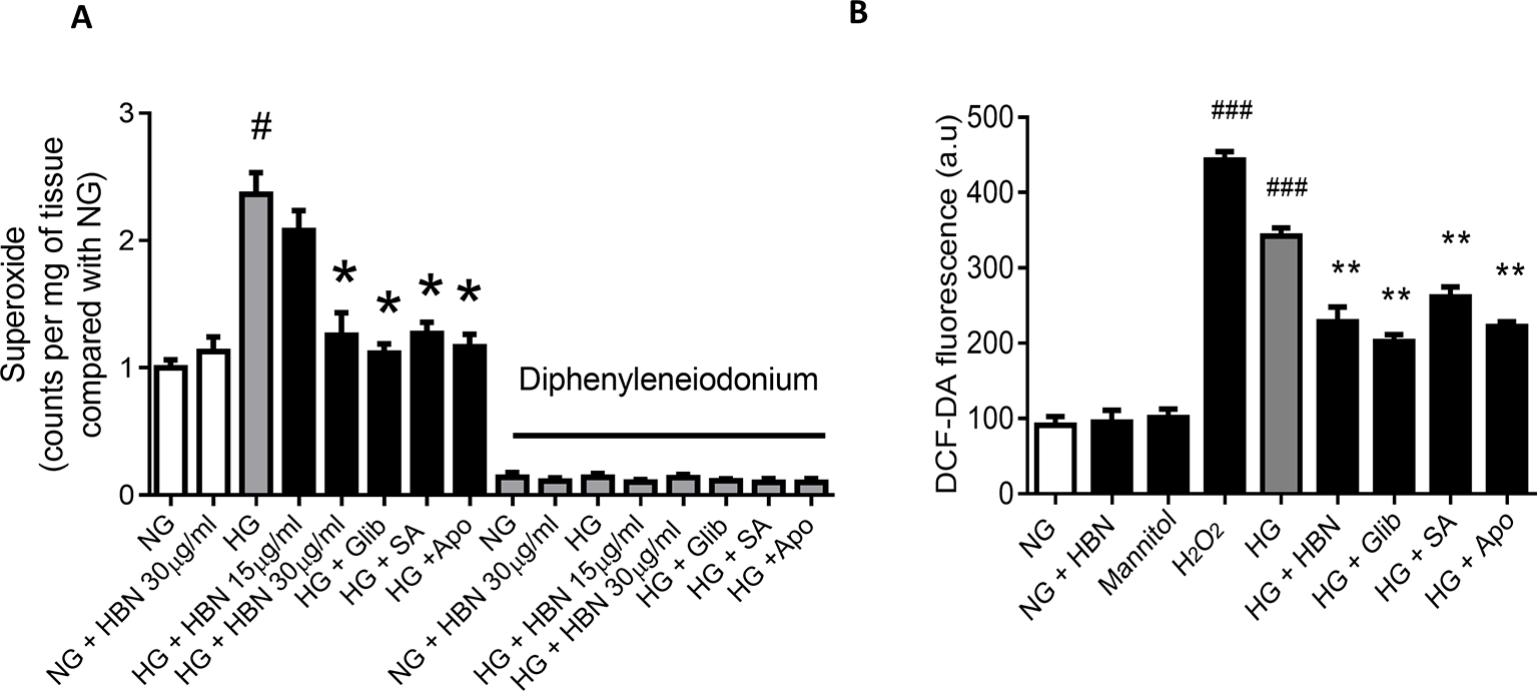
**(A)** Superoxide production measured by lucigenin-enhanced chemiluminescence assay in the aorta of C57BL/6J mice in the absence and presence of diphenyleneiodonium, NOX inhibitor and **(B)** level of intercellular ROS measured by DCF-DA assay after treatment with normal glucose (NG, 5 mM), high glucose (HG, 30 mM), mannitol (25 mM), H2O2 (200 µM), calcium ionophore (A23187, 5 µM), Hydrolysed bird nest (HBN, 30 µg/mL), sialic acid (SA, 20 µg/mL), glibenclamide (Glib, 10 µM) and apocynin (Apo, 20 µM) in HUVECs for 48 hours. Results are means ± SEM of 6 independent experiments. ^#^P < 0.05 and ^###^P < 0.001 compared with NG, *P <0.05 and ** <0.01 compared to HG.

HG produced high levels of vascular superoxide anions (**Figure 4A**) in isolated mouse aorta and intercellular ROS in HUVECs (**Figure 4B**) compared to NG, as measured by LEC and DCF-DA respectively. Co-treatment with HBN (30 µg/mL) significantly reduced the production of vascular superoxide anion and intercellular ROS in HUVECs compared to HG. Similarly, co-treatment with sialic acid (20 μg/mL), glibenclamide (10 μM), and apocynin (20 μM) reduced the vascular superoxide anion and intercellular ROS in HUVECs. The level of vascular superoxide anion and intercellular ROS in HUVECs in HBN alone in HG was similar to the NG-treated group.

### HBN Restored High Glucose-Induced Production of NO in HUVECs and NO Level in *db/db* Mice

**Figure 5:**
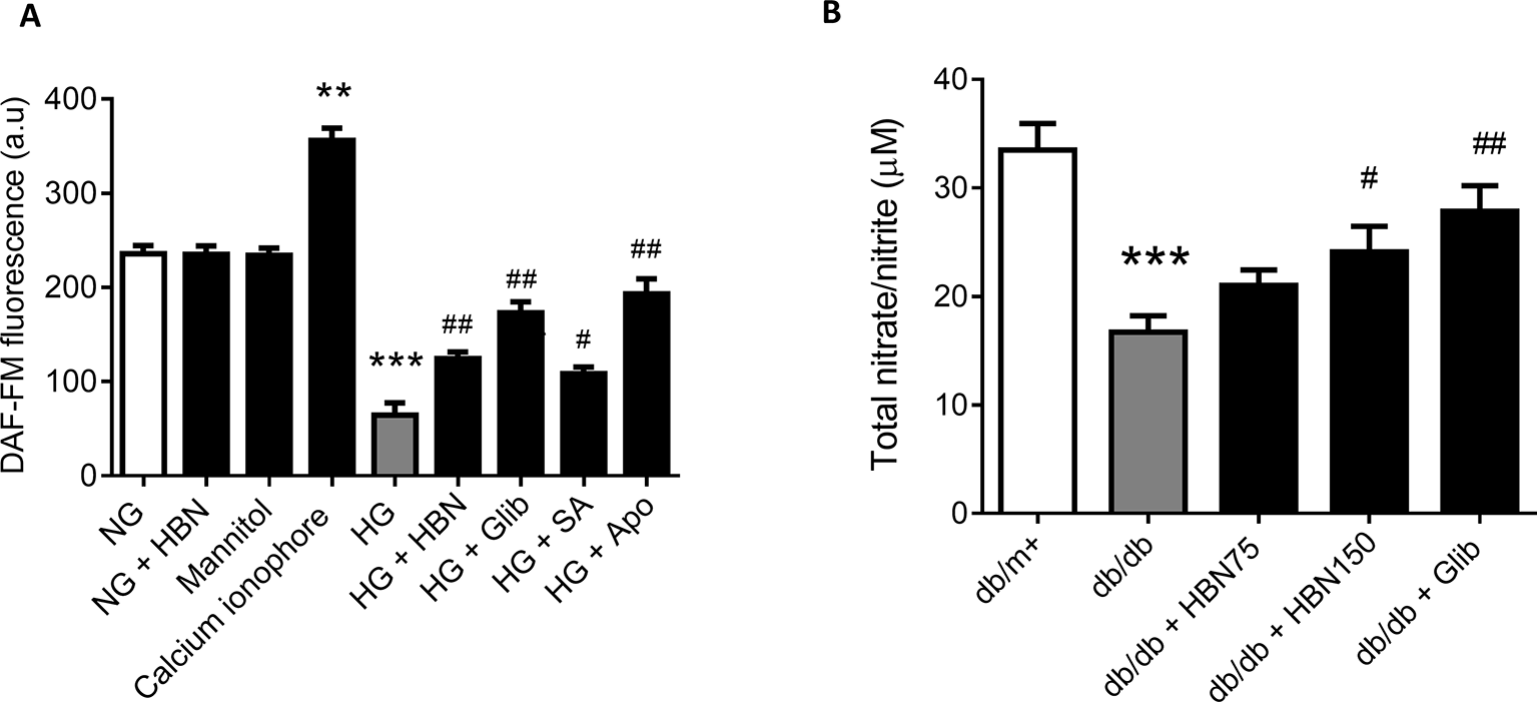
**(A)** Nitric oxide (NO) level as measured by DAF-FM after treatment with normal glucose (NG, 5 mM), high glucose (HG, 30 mM), mannitol (25mM), H2O2 (200 µM), calcium ionophore (A23187, 5 µM), hydrolyzed bird nest (HBN, 30 μg/mL), sialic acid (SA, 20 μg/mL), glibenclamide (Glib, 10 μM), and apocynin (Apo, 20 μM) in HUVECs for 48 h. **(B)** in serum of db/db mice treated with hydrolyzed bird nest (HBN, 75 and 150 mg/kg) and glibenclamide (1 mg/kg) for four weeks. Results are mean ± SEM of three experiments. **P < 0.01 and ***P < 0.001 compared to control; ^#^P < 0.05 and ^##^P < 0.01 compared to HG.

The reduced level of NO in HUVECs in response to HG was reversed by co-treatment with HBN (30µg/mL), sialic acid (20 μg/mL), glibenclamide (10 μM), and apocynin (20 μM). Meanwhile, the positive control, calcium ionophore (A23187) significantly increased the NO level. HBN and mannitol co-treatment did not affect NO level in the NG-treated aorta (**Figure 5A**).

The level of NO in *db/db* mice was depleted about 50% compared to the *db/m+* which demonstrated a reduction from 33.5 µM to 16.7 µM (**Figure 5B**). Treatment with HBN (150 mg/kg) increased the level of NO up to 24 µM compared to vehicle control. Meanwhile, the positive control, glibenclamide (1 mg/kg) significantly increased the NO level to 27.9 µM (**Figure 5B**). This results from *in vivo* study is in agreement with the *in vitro* study.

### HBN Inhibited High Glucose-Induced Oxidative Stress Associated Proteins in HUVECs and *db/db* Mouse Aorta

**Figure 6:**
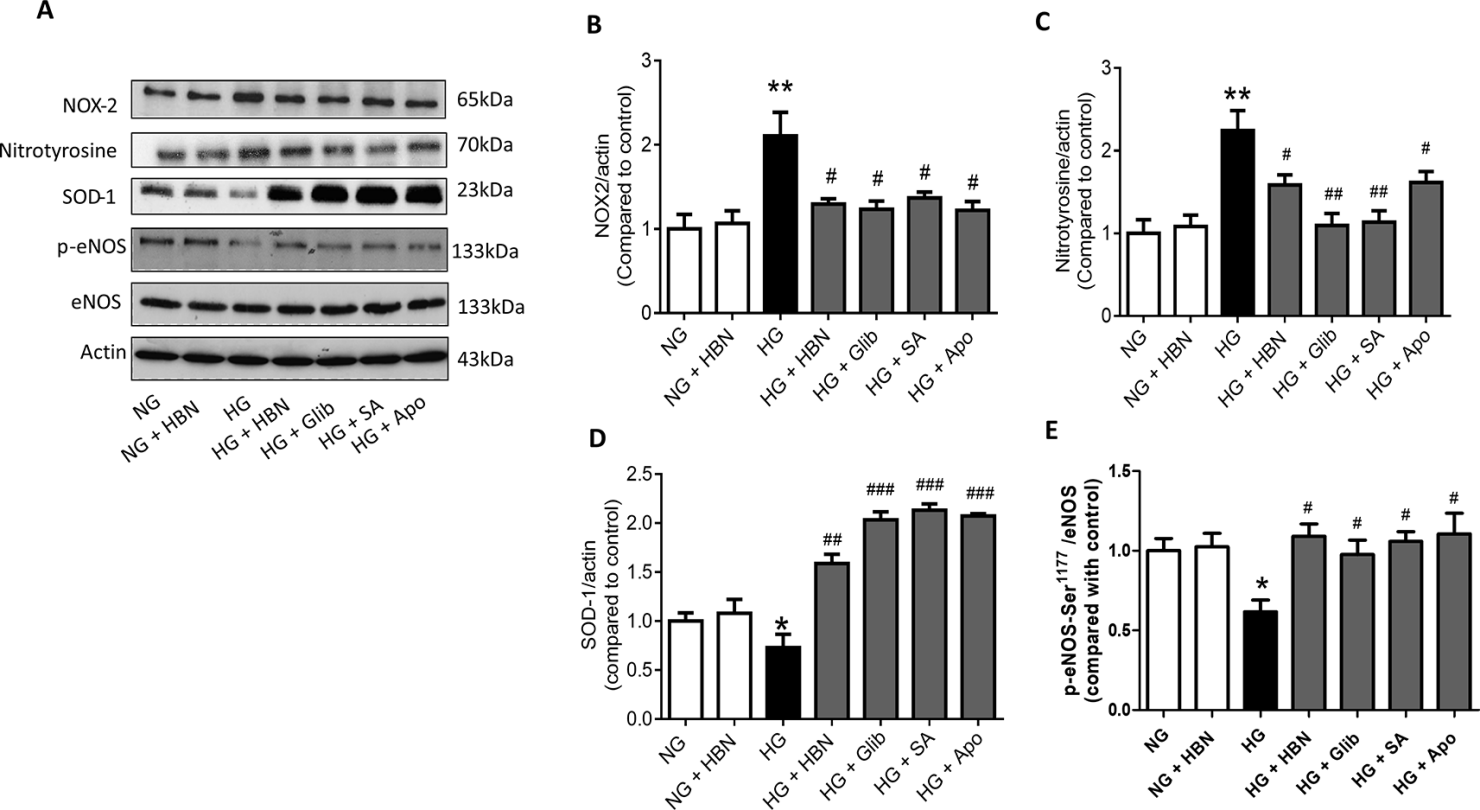
Western blot and quantitative data showing proteins in HUVECs treated with normal glucose (NG, 5 mM), high glucose (HG, 30 mM), hydrolyzed bird nest (HBN, 30 μg/mL), sialic acid (SA, 20 μg/mL), glibenclamide (Glib, 10 μM), and apocynin (Apo, 20 μM) for 48 h. Results are means + SEM of four separate experiments. *P< 0.05 and **P < 0.01 compared to control; ^#^P < 0.05, ^##^P < 0.01 and ^###^P < 0.001 compared to HG.

**Figure 7:**
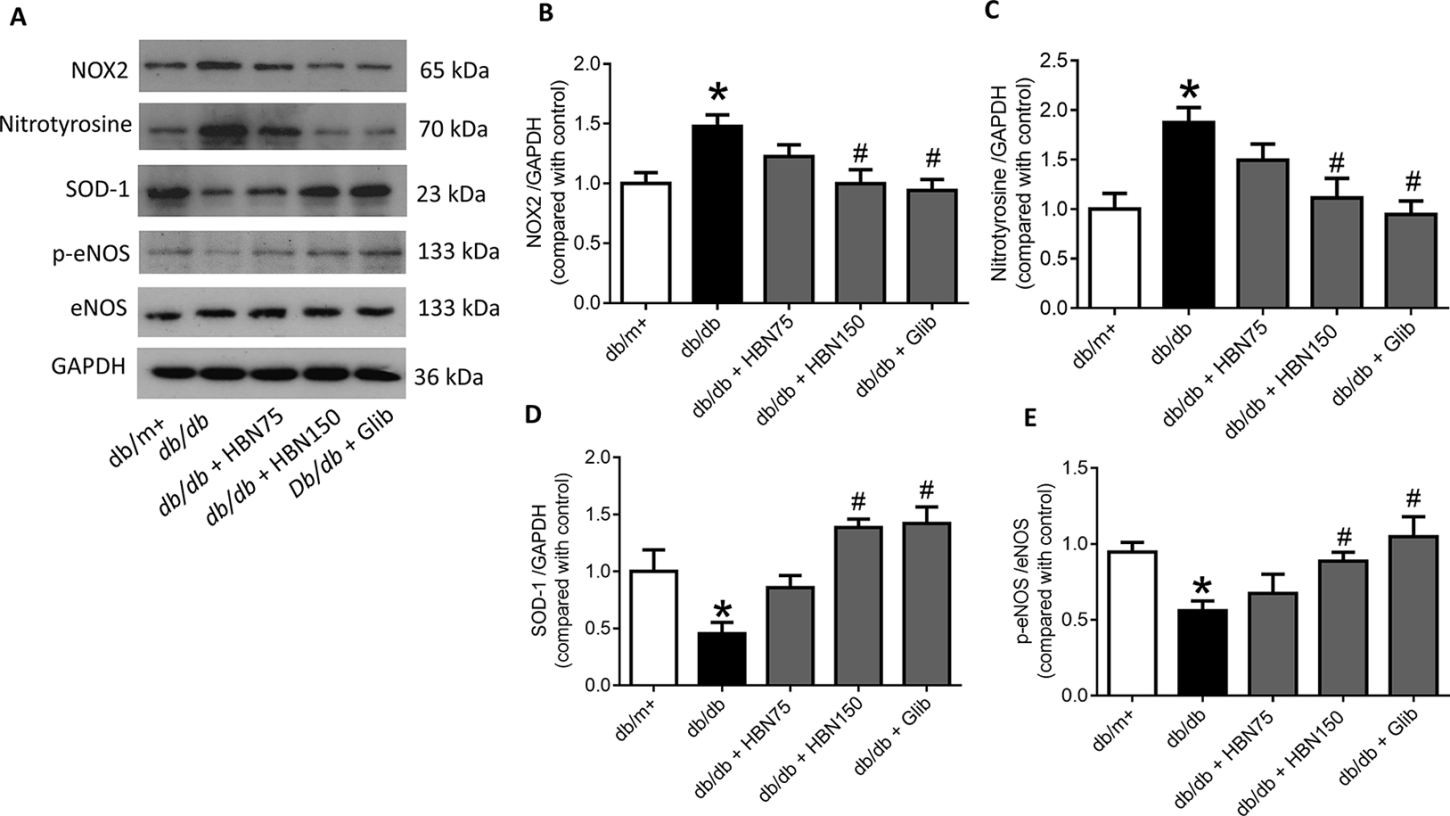
Western blot and quantitative data showing proteins in aorta of db/db mice treated with hydrolyzed bird nest (HBN, 75 and 150 mg/kg), and glibenclamide (1 mg/kg) for four weeks. Results are means + SEM of six separate experiments. *P< 0.05 compared to db/m+; ^#^P < 0.05 compared to db/db.

The effects of HBN were next explored on high glucose-induced oxidative stress associated proteins. NADPH oxidase 2 (NOX-2) and nitrotyrosine proteins were up-regulated in HG-induced HUVECs (**Figures 6A–C**) and in diabetic mouse aorta (**Figures 7A–C**). In contrast, the antioxidant protein (SOD-1) and eNOS activity were decreased in HG-induced HUVECs (**Figures 6A**, **D**, and **E**) and in diabetic mouse aorta (**Figures 7A**, **D**, and **E**). Co-treatment of HG with HBN (30µg/mL), sialic acid (20 μg/mL), glibenclamide (10 μM), and apocynin (20 μM) significantly reversed the elevated levels of NOX-2 (**Figure 6B**) and nitrotyrosine (**Figure 6C**) as well as increased the downregulated SOD-1 (**Figure 6D**) and p-eNOS (**Figure 6E**) protein levels induced by HG. No significant changes were observed between the NG and the NG + HBN group. Four-weeks treatment with 150 mg/kg HBN and 1 mg/kg glibenclamide to *db/db* mice significantly reversed the elevated vascular NOX-2 (**Figure 7B**) and nitrotyrosine (**Figure 7C**), while increased the reduced SOD-1 (**Figure 7D**) and p-eNOS (**Figure 7E**) protein levels compared to the *db/db* mice.

## Discussion

The present study provides experimental evidence that treatment with HBN effectively restored the impaired endothelium-dependent relaxations in mice aortas exposed to high glucose condition. In addition, the current work showed HBN reduced the expression of ROS markers (NOX-2 and nitrotyrosine), increased expression of SOD-1 and phosphorylated eNOS in HUVECs and aorta of *db/db* mice. These results indicate HBN treatment attenuates the hyperglycemia-induced endothelial dysfunction through the reduction in oxidative stress and increasing the NO bioavailability.

Increased oxidative stress and reduced nitric oxide (NO) bioavailability play a causal role in endothelial cell dysfunction occurring in the vasculature of diabetic patients (Rajendran et al., 2013). Prolonged exposure to high glucose *in vitro* or *in vivo* has been shown to inhibit ACh-induced endothelium-dependent relaxation, while not affecting SNP-induced endothelium-independent relaxation (Lau et al., 2013; Mangipudi and Hillier, 2013; El-Awady et al., 2014). A similar finding was also observed in the present study with 48-h incubation in HG and in aorta of *db/db* mice. Moreover, treatment with HBN significantly reversed the dysfunction demonstrating the vascular protective effect of HBN. The improvement demonstrated by HBN is comparable to apocynin, an antioxidant, and glibenclamide, an antidiabetic agent. High glucose has been shown to induce ROS production, which ultimately may contribute to the endothelial dysfunction (Cho et al., 2013; Salisbury and Bronas, 2015). Similarly, the vascular ROS, especially superoxide anion was elevated in aorta of *db/db* mice and aorta exposed to high glucose and HBN decreased the superoxide production in the high-glucose exposed tissues, indicating HBN protects against endothelial dysfunction by inhibiting ROS production. This is in agreement with previous researches that showed edible bird’s nest ameliorated oxidative stress by reducing production of ROS in SH-SY5Y cells and human keratinocytes (Yew et al., 2014; Hou et al., 2015; Lim et al., 2015).

In order to provide further insights into the mechanistic basis for the effects of HBN, the effect of HBN against high glucose-induced oxidative stress was investigated in aorta of *db/db* mice and HUVECs. Parallel to the finding with mice aorta, HUVECs exposed to high glucose also demonstrated an elevated level of ROS. This was accompanied by a decrease in NO level. ROS was mainly derived from NADPH oxidase which plays a role in the pathogenesis of vascular endothelial dysfunction in diabetes (Wong et al., 2010). High glucose-induced ROS elevation is mainly associated with increased expression of NADPH oxidase subunits such as NOX-2, which was also reduced by HBN. Basically, superoxide anions, the main species of ROS will react with NO to produce peroxynitrite radicals which will later lower NO bioavailability (Maritim et al., 2003). The role of peroxynitrite in high glucose-stimulated HUVECs and aorta of *db/db* was confirmed by detecting an increased expression of nitrotyrosine due to the facilitation of tyrosine nitration by peroxynitrate radicals. However, following co-treatment with HBN, expression of nitrotyrosine was significantly reduced. This was accompanied with increased production of NO as observed with an increase in p-eNOS expression and total nitrate/nitrite level in HBN treated *db/db* mice. Thus, the ROS inhibiting effect of HBN possibly contributed to restoring hemostatic imbalance which augments NO level in high-glucose stimulated endothelial cells in order to improve endothelial function.

Overall evidence suggests that on one hand, hyperglycemia induces free radicals; on the other hand, it impairs the endogenous antioxidant defense system in patients with diabetes (Pitocco et al., 2010). Endogenous antioxidant defense mechanisms such as glutathione (GSH), superoxide dismutase (SOD), and catalase (CAT), protects against toxic ROS (Gupta et al., 2014). HBN treatment increased SOD-1 protein level against the reduction induced by HG in the HUVECs and in treated *db/db* aorta. Likewise, previous reports by Hou et al. (2015) demonstrated that treatment with edible bird’s nest increased SOD activity and mRNA levels of SOD-1 in H_2_O_2_-induced oxidative stress in SH-SY5Y cells. Similarly, edible bird’s nest reduced the production of ROS in human HaCat keratinocytes and SH-SY5Y human neuroblastoma cells (Kim et al., 2012; Yew et al., 2014). HBN (Yida et al., 2015) and sialic acid (Pawluczyk et al., 2014) has been previously demonstrated to upregulate antioxidant enzymes including SOD at transcriptional levels. Although in the present study, only protein level of SOD was measured, the increase in gene expression is most likely reflected in the protein expression.

Previous study found that the composition on edible bird nest consist of protein (56.47–60.63%), water (17.26–24.05%), ash (3.29–7.41%), carbohydrates (1.04–2.48%), crude fiber (12.162.59%), and fat (0.07–12.57%) (Daud et al., 2016). The monosaccharides composition of glycoprotein in edible bird’s nest contains about 9% of sialic acid, 4.19–7.2% of galactosamine, about 5.3% of glucosamine, 5.03–16.9% of galactose, and about 0.7% of fructose (Kathan and Weeks, 1969). Wang and Brand-Miller (2003) reported that edible bird’s nest contains a high amount of sialic acid and this may contribute to brain development and learning ability. This is further supported by a recent work by Oliveros et al. (2018), whereby they demonstrated supplementation of sialic acid and sialylated Oligosaccharide supplementation during lactation improved learning and memory in rats. Similarly, sialic acid has been shown to restore mitochondrial SOD mRNA expression and quench oxidative burst in puromycin aminonucleoside-induced desialylation and oxidative stress in human podocytes (Pawluczyk et al., 2014). In 2016, Guo et al. has demonstrated exogenous supplement of sialic acid ameliorated atherosclerosis in apolipoprotein E-deficient mice partly by elevating antioxidant activity by restoring the activity or improving protein expression of antioxidant enzymes, thus demonstrating the beneficial effect of sialic acid on cardiovascular disease (Guo et al., 2016). The efficacy of HBN against high glucose-induced oxidative stress and endothelial dysfunction was comparable to the sialic acid, the main carbohydrate found in edible bird’s nest. Therefore, sialic acid contained in edible bird’s nest could be used as an important parameter for determining for their quality and their biological activities. The results suggest that sialic acid may represent the active compound in edible bird’s nest which is responsible for the mechanism involves reducing oxidative stress.

## Conclusion

In summary, both *in vitro*, *ex vivo*, and *in vivo* treatments with HBN significantly protect against high-glucose induced endothelial dysfunction by inhibiting oxidative stress and increasing NO bioavailability. These results provide further evidence for edible bird’s nest to be used as a functional food for the prevention of cardiometabolic diseases by combating oxidative stress and thus subsequently protect endothelial function in hyperglycemic conditions.

## Data Availability Statement

The datasets generated for this study are available on request to the corresponding authors.

## Ethics Statement

The study protocol involving the use of animals in the present study was approved by the University of Malaya Animal Care and Ethics Committee (Ethics Reference No: 2015-180709) and accredited by the Association for Assessment and Accreditation of Laboratory Animal Care International (AAALAC). The animal study was carried out in strict accordance with the established institutional guidelines and the NIH guidelines on the use of experimental animals. Consent to participate was not applicable.

## Author Contributions

MM, DM and KC participated in designing the study. ZZ and KC performed the *in-vitro*, *ex-vivo* and *in-vivo* experiments and analyzed the data. NZ, MC and YL performed the phytochemical analyses. DM and ZZ prepared the first manuscript draft. MM, KC and YL participated in editing and preparation of the final manuscript draft. All authors read and approved the final manuscript.

## Funding

This study was funded by Government Agency grant GA001-2017. The funding agencies played no role in the design of the study and collection, analysis, and interpretation of data and in writing the manuscript, which are fully the responsibilities of the authors.

## Conflict of Interest

The authors declare that the research was conducted in the absence of any commercial or financial relationships that could be construed as a potential conflict of interest.
